# ROS and RNS Alterations in the Digestive Fluid of *Nepenthes* × *ventrata* Trap at Different Developmental Stages

**DOI:** 10.3390/plants11233304

**Published:** 2022-11-29

**Authors:** Agnieszka Wal, Pawel Staszek, Barbara Pakula, Magdalena Paradowska, Urszula Krasuska

**Affiliations:** Department of Plant Physiology, Institute of Biology, Warsaw University of Life Sciences-SGGW, 02-776 Warsaw, Poland

**Keywords:** nitric oxide, superoxide anion, carnivorous plants, pitcher plants, plant digestion

## Abstract

The carnivorous pitcher plant, *Nepenthes* × *ventrata* (Hort. ex Fleming = *N. ventricosa* Blanco × *N. alata* Blanco), produces passive traps containing digestive fluid. Although reactive oxygen species (ROS) in the fluid were detected in some pitcher plants, the participation of reactive nitrogen species (RNS) in the digestion process has not yet been examined. The aim of this work was to investigate the production of superoxide anion (O_2_^•−^), nitric oxide (NO) and peroxynitrite (ONOO^−^) levels in the digestive fluid of traps throughout organ development. We revealed the ROS and RNS occurrence in the digestive fluid, linked to the ROS-scavenging capacity and total phenolics content. In digestive fluid from the fed traps, NO emission was higher than in the fluid from the developed unfed pitcher. The concentration of nitrite (NO_2_^−^) decreased in the fluid from the fed traps in comparison to the unfed ones, pointing at NO_2_^−^ as the key source of NO. The enhanced emission of NO was associated with lowered content of ONOO^−^ in the fluid, probably due to lower production of O_2_^•−^. At the same time, despite a decline in total phenolics, the maximum ROS scavenging capacity was detected. In addition, ROS and RNS were noted even in closed traps, suggesting their involvement not only in digestion per se but also their action as signaling agents in trap ontogeny.

## 1. Introduction

Carnivorous plants (*Plantae carnivorae*) are able to attract prey, kill it, digest the body, and absorb released nutrients. This is a relatively unusual method of nourishment for autotrophs, described as the “carnivorous syndrome”. This system of obtaining nutrients from other organisms has made these creatures known as “the most wonderful plants in the world” [1,2]. Carnivorous plants do not have one ancestor and evolved independently, several times, in different parts of the world [2], and they are “the products” of an imposed adaptation to a particular environment. Carnivorous plants occur in specific habitats, mostly with moist conditions and low nutrient availability, such as swamps or peat bogs with waterlogged, poorly aerated and acidic soil [3,4]. The traps of carnivorous plants are metabolically active organs that evolve from “typical” leaves. Due to their ability to perform certain movements, traps are divided into categories: sticky, adhesive traps (fly-traps), pitcher-shaped containers, moveable snap traps, and suction bladders or eel traps [5]. The modified leaves have several features facilitating the attraction of potential prey: specific shapes, particular colors, and UV-visible patterns. Moreover, they can liberate volatiles and produce nectar [6,7,8].

Pitcher plants that form pitfall-shaped traps include: Sarraceniaceae, Cephalotaceae, and Nepenthaceae [9]. *Nepenthes* (Nepenthaceae) converted leaves into jug-shaped containers to lure and trap arthropods and other small animals [10]. It is currently estimated that the genus *Nepenthes* contains over 140 species [11]. These tropical plants are found mainly in Indonesia. Some species also occur in India (*N. khasiana* Hook. f.), Sri Lanka (*N. distillatoria* L.), Seychelles (*N. pervillei* Blume), and Madagascar (*N. madagascarensis* Poir. and *N. masoalensis* Schmid-Hollinger) [12,13]. Their habitats are mostly heath forests (*kerangas*), peat-swamp forests, and mountainous forests [14]. *Nepenthes*’ growth and development are usually related to the formation of dimorphic traps on the tips of their leaves: “terrestrial” and “aerial” pitchers [14,15]. The first type of trap is present mostly in young plants, which form compact rosettes and straight tendrils tipped with ovary pitchers. Mature plants, characterized by climbing stems with long internodes and curled tendrils, form “aerial” pitchers [14]. Recently, a new type of pitcher was discovered for *N. pudica*—underground traps [16]. The typical *Nepenthes* trap is divided into three zones [17], which include: a peristome (ribbed upper rim of the pitcher), a waxy zone [18], and a digestive zone [19]. A lid is considered as part of the trap, which mainly protects the trap against rain droplets [18,20].

Digestion occurs in the presence of different proteins, including Nepenthesins 1 and 2, specific aspartic proteases with broad cleavage specificity, high thermal stability, and optimal activities at acidic pH [21]. Other proteins that participate in digestion are phosphatases, chitinases, and esterases or peroxidases of class III (POx). Apart from the main function of the hydrolysis of organic compounds, some of these proteins take part in the host defense against microorganisms, their competitors for released nutrients. This function is attributed to POx, which generates reactive oxygen species (ROS). By contrast, this enzyme may also function as an antioxidant (through the reduction of hydrogen peroxide (H_2_O_2_) using electrons from different donors, including phenolic compounds (PCs) [22]. The nutrient absorption is achieved at the bottom of the trap equipped with a permeable cuticle and glands, which produce digestive enzymes. The transporters located across the pitcher wall are responsible for the active transport of liberated nitrogen into plant tissues [23]. The presence of thaumatin-like proteins belonging to pathogenesis-related proteins and POx has been demonstrated in *Nephenthes* traps. Additionally, some other compounds have been noted in digestive fluid, including naphthoquinones [24] and flavonols [25]. More than 15 years ago, there was a report regarding the first step of digestion in the pitcher fluid of *N*. *gracilis* Korth., which is accompanied by the generation of free radicals, mostly hydroxyl radicals (^•^OH) [26].

The incomplete reduction or excitation of oxygen leads to ROS generation [27]. The physiological activity of these compounds is dependent on their concentration. In non-stress conditions, the functionality of organisms is dependent on a low level of ROS (acting as signaling components) [28]. Furthermore, ROS participate in the removal of pathogenic microbiomes [29]. Uncontrolled ROS production or inefficient antioxidant-system activity lead to the generation of ROS at toxic levels. This increased ROS content is related to oxidative stress [30]. Commonly, the ROS family includes superoxide anion (O_2_^•−^), as well as ^•^OH and H_2_O_2_, which are produced in plant tissues, mainly in chloroplasts, mitochondria, peroxisomes, and in the apoplast [31]. The reactivity of ROS depends on its radical/non-radical nature [32,33,34]. ROS react with the basic cellular molecules, including proteins and nucleic acids [28,32]. They modify amino-acid residues in peptides/proteins, leading to the modification of protein structure and function. The irreversible oxidation (e.g., carbonylation) of peptides makes them prone to faster degradation [28]. The increase in the activity of proteases was noted after the reaction of free radicals with proteins [35]. Cellular ROS balance is under the regulation of the ROS antioxidant system, which is divided into enzymatic and non-enzymatic. The enzymes that modulate ROS levels included superoxide dismutase (SOD), catalase (CAT), different peroxidases, and glutathione reductase (GR). The best-known low-molecule antioxidants are the reduced form of ascorbic acid (ASA) and the reduced form of glutathione (GSH), [32,36,37]. POxs can be exported to the extracellular space and are involved in ROS metabolism (formation or elimination) [22]. Furthermore, many of the known PCs can directly or indirectly interact with ROS. These endogenous growth regulators can chelate copper and iron ions and, thus, they are able to reduce free-radical formation due to the Fenton reaction [38].

Reactive nitrogen species (RNS) are products of nitric oxide (NO) and their reaction with oxygen or O_2_^•−^. NO’s reaction with the latter molecule leads to the formation of peroxynitrite (ONOO^−^), a potent oxidizing and nitrating agent. The best-known RNS are: NO radical form (^•^NO), nitrosonium cation (NO^+^), nitroxyl anion (NO^−^), and ONOO^−^ [39,40]. RNS are produced in various cellular compartments, including apoplastic space] [39,41,42]. Similarly to ROS, their action depends on concentration and location; they serve as regulatory or signaling molecules but are also cytotoxic (high level of RNS) [30,40,41]. In plants, NO synthesis depends on the reduction or oxidation pathways, and can be formed by enzymatic and non-enzymatic reactions. Nitrite (NO_2_^−^) is the key donor of various RNS. Under low pH, the protonation of NO_2_^−^ leads to nitrous acid (HNO_2_) formation; subsequently, the formation of different nitrogen oxides (NOx) depends on the actual redox state of the local environment and the presence of molecular oxygen or ROS [43,44]. Reductants increase the rate of NO generation from NO_2_^−^ [44]. RNS react with nucleic acids, fatty acids or proteins [45]. Nitrated proteins (molecules with irreversible modified tyrosine’s or tryptophane’s residues) are also believed to be degraded faster, as is observed for carbonylated proteins [46,47].

It is known that ROS may be liberated outside plant tissues, including the digestive fluids of pitcher plants [26,48]. On the other hand, the emission of NO from autotrophs was studied in nitrogen-supplied plants. NO_2_^−^ application in the growing medium of examined plants stimulated NO liberation even in darkness [49]. No data indicate the presence of NO in the digestive fluid of pitcher plants. Furthermore, little is known about the content of NO_2_^−^, which may be the main source of various RNS at acidic pH during digestion; the process carried out in the trap.

The aim of this work was to determine the alterations in ROS levels and RNS production in the digestive fluid of *Nepenthes* × *ventrata* throughout their organ development until their aging.

We hypothesize that ROS and RNS act as signaling molecules that regulate development of the trap and digestion process. We propose the presence of ROS–RNS crosstalk in digestion fluid, from the trap’s formation until the trap’s ageing.

## 2. Results

### 2.1. O_2_^•−^ Generation in Digestive Fluid

**Figure 1:**
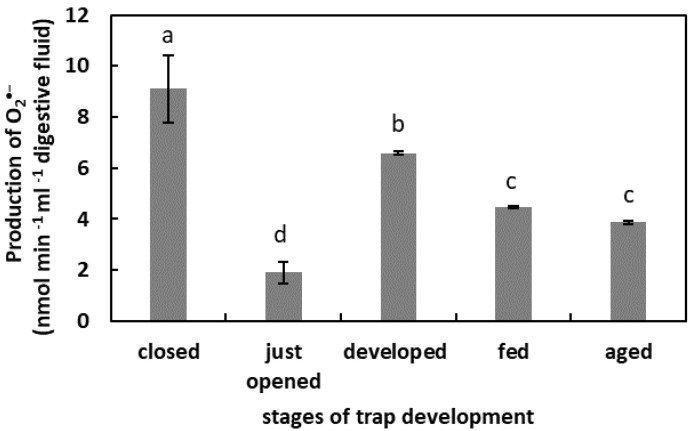
Superoxide anion (O_2_^•−^) production in digestive fluid in the traps at different developmental stages: closed, just opened, developed, fed (one day after feeding), and aged. Values are average ± SD of 3 repetitions. Letters (a–d) indicate homogenous groups determined after ANOVA and post hoc Fisher’s LSD test at *p* ≤ 0.05.

The highest level of O_2_^•−^ production was in the digestive fluid of the closed traps (9 nmol min^−1^ mL^−1^) (Figure 1). In the developed traps, the O_2_^•−^production was 6 nmol min^−1^ mL^−1^. In the fed traps and in the aged traps, the O_2_^•−^ production in fluid was at similar levels. In the digestive fluid of the just-opened traps, the O_2_^•−^ generation was the lowest.

### 2.2. The Free-Radical-Scavenging Capacity in Digestive Fluid

**Figure 2:**
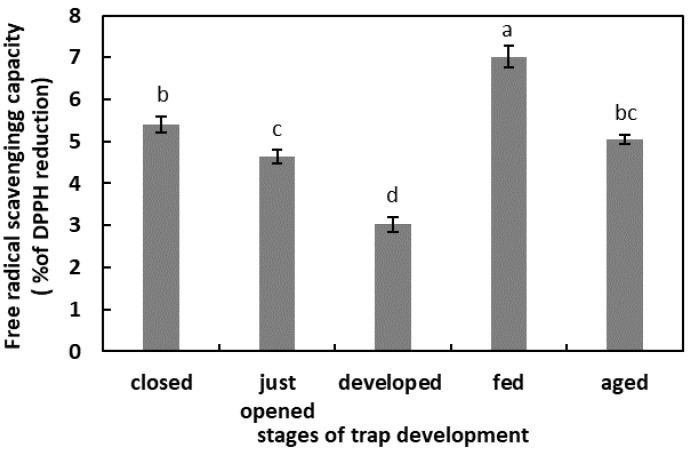
Total capacity of the free-radical reduction in digestive fluid in the traps at different developmental stages: closed, just opened, developed, fed (one day after feeding), and aged. Values are average ± SD of 3 repetitions. Letters (a–d) indicate homogenous groups determined after ANOVA and post hoc Fisher’s LSD test at *p* ≤ 0.05.

In the unfed, developed traps, the total capacity of the free-radical reduction was the lowest. One day after feeding, the total free-radical-scavenging capacity was the highest in the studied model. In the digestive fluid of the closed traps, just-opened traps, and aged traps, the total capacity of the free-radical reduction was at a similar level, of about 5% (Figure 2).

### 2.3. The Phenolic Compounds (PCs) Content in Digestive Fluid

**Figure 3:**
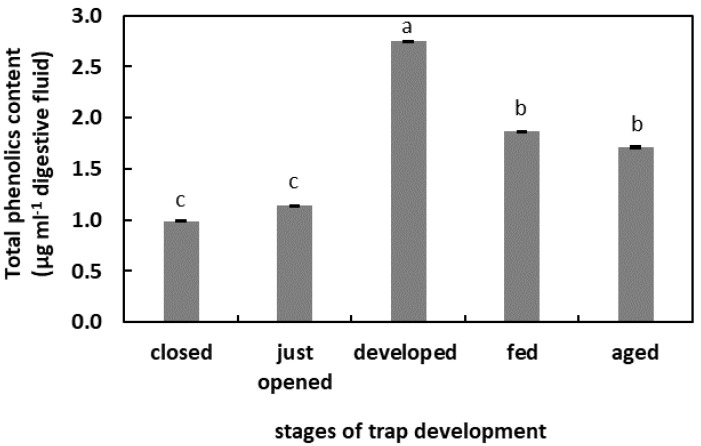
Phenolic compounds (PCs) content in the digestive fluid in the traps at different developmental stages: closed, just opened, developed, fed (one day after feeding), and aged. Values are average ± SD of 3 repetitions. Letters (a–c) indicate homogenous groups determined after ANOVA and post hoc Fisher’s LSD test at *p* ≤ 0.05.

In the digestive fluid of the closed and just-opened traps, the PCs content was at similar levels (below 1.4 µg mL^−1^ digestive fluid). An increase in the PCs content was observed in the digestive fluid taken from the unfed developed traps. This increase was more than 2.5 times that observed in the closed traps. The total PCs content in the digestive fluid of the fed traps and aged traps was at similar levels, about 1.8 µg mL^−1^ digestive fluid (Figure 3).

### 2.4. The Generation of NO in Digestive Fluid

**Figure 4:**
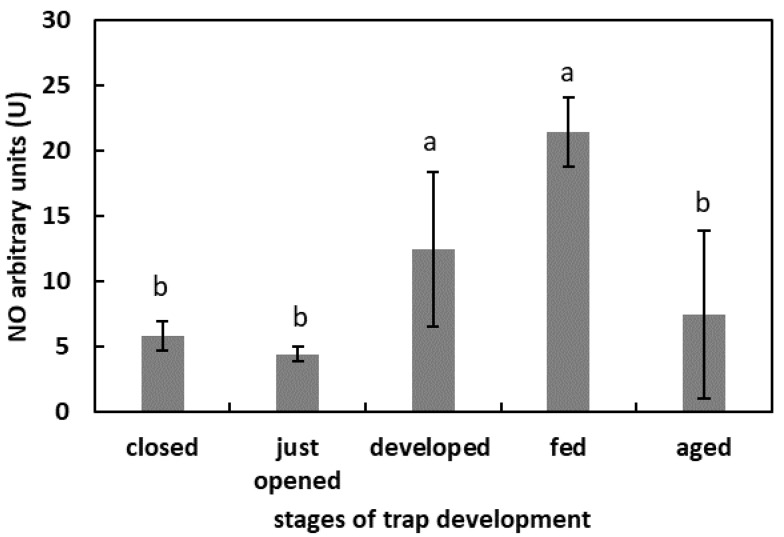
NO generation in digestive fluid in the traps at different developmental stages: closed, just opened, developed, fed (one day after feeding), and aged. Values are average ± SD of 3 repetitions. Letters (a,b) indicate homogenous groups determined after ANOVA and post hoc Fisher’s LSD test at *p* ≤ 0.05.

One day after feeding, the fluorescence of the DAF-FM was the highest in the studied model (more than 20 U) (Figure 4) The lowest fluorescence signal, without significant differences, was in the digestive fluid of the closed traps, freshly opened traps, and aged traps (around 5 U). In the developed traps that were not artificially fed, the fluorescence of the DAF-FM was 12.4 U.

### 2.5. The Generation of ONOO^−^ in Digestive Fluid

**Figure 5:**
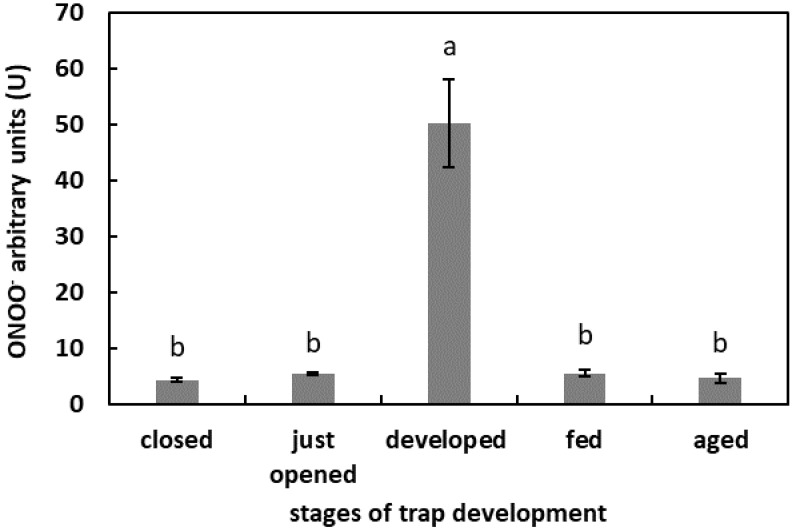
ONOO^−^ generation in digestive fluid in the traps at different developmental stages: closed, just opened, developed, fed (one day after feeding), and aged. Values are average ± SD of 3 repetitions. Letters (a,b) indicate homogenous groups determined after ANOVA and post hoc Fisher’s LSD test at *p* ≤ 0.05.

The highest fluorescence of APF was in the digestive fluid of the developed but unfed traps (50.2 U). In the digestive fluid of the closed, freshly opened, fed, and aged traps, the fluorescence of APF was at a similar level, of more than 5 U but below 10 U (Figure 5).

### 2.6. NO_2_^−^ Content in Digestive Fluid

**Figure 6:**
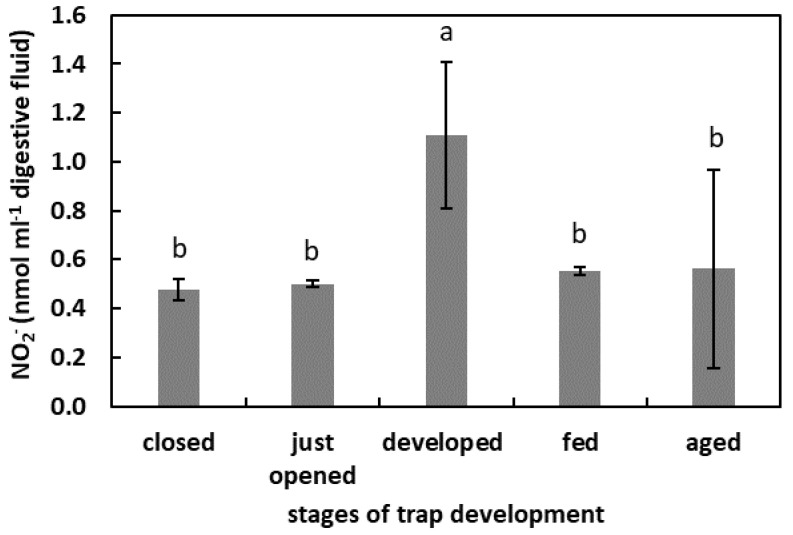
The NO_2_^−^ concentration in digestive fluid in the traps at the different developmental stages: closed, just opened, developed, fed (one day after feeding), and aged. Values are average ± SD of 3 repetitions. Letters (a,b) indicate homogenous groups determined after ANOVA and post hoc Fisher’s LSD test at *p* ≤ 0.05.

In the digestive fluid of the developed traps, the NO_2_^−^ concentration was the highest (1.1 nmol mL^−1^ digestive fluid). The NO_2_^−^ concentration in the digestive fluid of closed, just opened, fed, and aged traps was at a similar level, about 18 µg mL^−1^ digestive fluid (Figure 6).

## 3. Discussion

The natural environment of carnivorous plants is characterized by the poor availability of minerals in the rhizosphere. To survive, they have developed extremely modified “typical” leaves, which, in a way, replaced the roots in the function of absorbing nutrients. Pitcher plants, including *Nepenthes* of different species, developed pitfall traps; the fluid, which fills the lower part of the trap, is a specific mixture that enables the release of nutrients from the bodies of the captured prey [11,23].

ROS are indispensable products of plant cellular metabolism. As signaling molecules, ROS are known for their role in abiotic- and biotic-stress-related reactions. However, they are also involved in numerous processes throughout the plant ontogeny, from embryogenesis and germination through to root, shoot, and flower development.

Apoplastic ROS production is known to have an important regulatory effect [22]. Moreover, ROS are liberated outside the tissues, as was demonstrated for germinated seeds. The germination of radish (*Raphanus sativus* cv Eterna) seeds was accompanied by ROS and POx release [48]. The authors propose that this ROS generation is a developmentally controlled, protective mechanism against pathogen attack. The release of H_2_O_2_ into the germination medium of apple (*Malus domestica* Borkh.) embryos and from tomato (*Solanum lycopersicum* L.) seedlings was also shown [50,51], pointing to the universality of this phenomenon in the plant kingdom. The presence of ROS in the digestive fluid of carnivorous plants has been confirmed. An et al. [52] demonstrated that among the compounds isolated from the pitcher fluid of *N. alata* Blanco are the digested products of the exogenously oxidized proteins. Subsequently, Chia et al. [26] confirmed the presence of ^•^OH in *N. gracilis* Korth. digestive fluid. The authors proposed that this free radical initiates and facilitates the digestion of proteins or peptides. ROS involvement in proteolysis has been shown as protein degradation is facilitated by irreversible oxidation, mostly carbonylation [28,53]. The results of our research confirmed the presence of ROS in radical form in digestive fluid during the whole trap ontogeny, even in the closed organs. As this phase of trap development is not related to digestion, their function may be associated with the following: the facilitation of the growth of the trap tissues; the oxidation of the fluid compounds before opening the organ to modify certain compounds, for instance, make them inactive or to alkalinize the pH, in order to prevent the activity of some pH-dependent enzymes. It has been demonstrated for some *Nepenthes* plants that the secreted fluid is bacteria-free and unsuitable for microbial growth [54]. Hence, the ROS could serve as an antibacterial agent. A highly oxidized environment can also be provided by the chlorine ions detected in the fluid of closed *N. alata* pitchers [54]. By contrast, in just-opened traps, the free-radical content strongly decreased. We assume that at this stage of trap development, it is likely that the composition of the microbiome changes [55]. An environment with strong oxidizing properties could negatively affect the growth of beneficial microorganisms. The subsequent phases of the trap’s existence are associated with an increase in the ROS content until digestion. This higher ROS level may be due to the higher pH of the fluid, as pH alkalization stimulates the production of some ROS, as was demonstrated for mitochondrial ROS production [56]. The decomposition of prey bodies is linked to a slight decrease in ROS level (as the pH lowers). This physiological phase is also associated with an increase in free-radical-scavenging capacity. To avoid toxic ROS overaccumulation (e.g., plant responses to the mechanical stimuli by the caught prey), antioxidants modulate the free-radical content, and we propose that PCs play an important role in this process. The concentration of the total PCs was lower as digestion proceeded. The activity of PCs in plant responses to different stressors, including biotic stressors, is well known [57]. As substrates, PCs are also used by POxs in peroxidative cycle to reduce H_2_O_2_ content [22]. We propose that PCs may be used by POx to reduce ROS levels, as demonstrated for Arabidopsis’ increased POx activity in response to pathogen attack [58]. The POx activity should be experimentally verified in the pitcher fluid of *N. ventrata*, although a higher level of PCs in the mature but not artificially fed traps may point to the protective role of these compounds in plant–microbe interactions. Some of these compounds may also be of microbial origin [57].

There are no research data that confirm the formation, liberation or generation of NO in pitcher fluid, especially during the digestion process. Our results demonstrated for the first time that RNS are present in *N. ventrata* trap fluid. The presented data strongly suggest that the digestion process is accompanied by increased RNS generation (detected as NO formation using the fluorescence method) in the pitcher fluid. Moreover, during trap development, changes in RNS level were observed. The lowest NO formation was noted in the closed traps and the just-opened traps. Nevertheless, the NO is clearly of plant origin. The question that arises is what the NO function is at the very beginning of the functioning of traps. The mature, opened, but unfed traps was characterized by higher NO levels in the trap fluid than in the fluid of immature or closed traps. The function of NO remains unknown. High NO levels in the fluid during digestion strongly points to the involvement of RNS in this process. We propose two roles of RNS. The first is linked to the ROS modulation by NO; the second is linked to the RNSs’ action as protein modifiers. We also do not exclude the involvement of RNS- or NO^−^-modified molecules in long-distance signaling in the course of prey digestion. By analogy with the action of ROS, the modifications of proteins of a reversible or non-reversible nature fulfill two functions: the modification of the target protein activity and the stimulation of proteolysis. However, the question of whether NO formation after opening the trap is due to the plant’s activity or to the activity of microorganisms inside the trap requires further investigation. The release of NO from plants into the surrounding environment as a response to different stimuli (e.g., light) was confirmed [49]. It has been proposed that NO emission, especially in darkness (light-exposure limitation) may be increased by NO_2_^−^ addition, and this is a physiological reaction common to all plants [49]. Our results indicated differences in NO_2_^−^ content in the digestive fluid. Firstly, very low levels of these ions were detected in the closed and just-opened traps. These findings are in agreement with data concerning trace amounts of nitrate (NO_3_^−^) in the fluid of closed *N. alata* pitchers [54]. Furthermore, the highest level was noted for the mature but unfed traps. A decrease in NO_2_^−^ content was characteristic for the fed traps, after one day of digestion. These results suggest that the major source of NO in the digestive fluid may be NO_2_^−^. The protonation of NO_2_^−^ occurs under low pH (similarly to the human stomach), leading to the formation of dinitrogen trioxide (N_2_O_3_), a strong nitrosating agent (equations below). Further NO and radical nitrogen dioxide (NO^•^_2_) are formed in the reaction of disproportionation. This NO formation requires both high NO_2_^−^ concentration and low pH [59].

2NO_2_^−^ + 2H^+^ ⇌ N_2_O_3_ + H_2_O

N_2_O_3_ ⇌ NO^•^_2_ + NO

The question arises as to the source of NO_2_^−^. We propose that it is of both plant and prey origin. NO_2_^−^ may be formed from NO (generated in plant tissues or produced by microbes), which reacts with oxygenated hemoproteins in a process called NO deoxygenation, catalyzed by hem-containing proteins, including hemoglobins [59], which are also present in plants [60]. These results are linked to those obtained after the measurement of ONOO^−^ formation in pitcher fluid. The lowest ONOO^−^ levels were noted for the closed and just-opened traps, corresponding to the lowest NO emission and NO_2_^−^ content in the analyzed fluid at these developmental stages of the organ. As ONOO^−^ is a strong nitration agent, it may interfere with the formation of a relationship with microorganisms, or it may modify necessary enzymes and other proteins involved in prey digestion. The highest levels of NO_2_^−^, the intense generation of O_2_^•−^, and ONOO^−^ formation were characteristic of a fully mature trap “ready” to catch prey. By contrast, even if digestion is accompanied by high NO formation in the digestion fluid, the NO_2_^−^ content and the ONOO^−^ formation are decreased. This points to the presence of strongly regulated NO biochemistry in digestive fluid during prey decomposition.

The ageing of traps is the last developmental stage along with the decrease in ROS and RNS formation, even as the NO_2_^−^ level increases. It can be assumed that this tissue acts as a nutrient donor before abscission from the plant. High concentrations of NO_2_^−^ at this stage are probably not linked to NO generation but, instead, they may protect against pathogen growth and mold development. Nevertheless, further research should be undertaken to confirm this hypothesis.

## 4. Materials and Methods

### 4.1. Plant Culture

**Figure 7:**
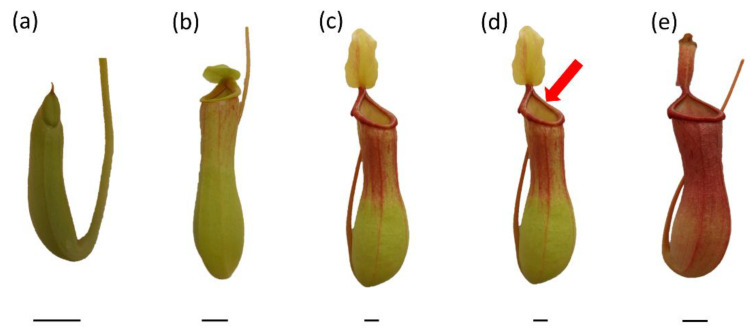
Traps of *Nepenthes* × *ventrata* at various stages of development: closed trap (**a**), just-opened trap (**b**), fully developed, non-artificially fed trap (**c**), fully developed trap artificially fed with two fruit flies (*Drosophilla melanogaster*) or egg-white solution (**d**), and aged trap (**e**). Bar = 1 cm.

Mature pitcher plants *Nepenthes* × *ventrata* Hort. ex Fleming (=(*N. ventricosa* Blanco × *N. alata* Blanco)) were used as plant materials. Plants were grown in a greenhouse under conditions of high humidity (60%), controlled temperature (28 °C), and natural light (spring–summer season). Plants were grown in a mixture of acid peat and perlite with sphagnum moss, and they were watered with rainwater every other day. After opening, pitcher-trap inlets were covered with a single layer of sterile gauze to control prey intake. For experiments, traps at various stages of development were selected: closed traps (Figure 7a), just-opened traps (up to 16 h after opening) (Figure 7b), fully developed, non-artificially-fed traps (Figure 7c), fully developed traps, one day after artificial feeding with two fruit flies (*Drosophilla melanogaster*) (live flies were placed in a fluid of the trap using forceps) or egg-white solution (Figure 7d), and traps with visible symptoms of ageing (browning of the tissues of the lid) (Figure 7e). To measure the generation of O_2_^•−^, the NO_2_^−^ content, the free-radical-scavenging capacity and the phenolic-compound content of traps artificially fed with egg-white solution, 40 µg of egg-white solution (1 µg^−1^ µL) was added to the pitchers. To measure NO and ONOO^−^ generation, traps were fed with fruit files. Each of developmental type of trap was examined on separate plants. The digestive fluid was collected and used for analyses; after short centrifugation (5522× *g*, 5 min at 4 °C), the obtained supernatant was transferred to the sterile tubes for further analyses.

### 4.2. Measurement of O_2_^•−^ Generation in Digestive Fluid

Measurement of oxidation of epinephrine by O_2_^•−^ was conducted according to Misra and Fridovich [61]. Digestive fluid (2 mL) was mixed with 1 M Tris-HCl pH 7.5 (0.5 mL) to buffer the sample. Next, the sample (585 µL) was mixed with 1 M Tris-HCl pH 7.5 (15 µL), and 300 µL of 60 mM epinephrine dissolved in 50 mM HCl was added. The oxidation of epinephrine to adrenochrome was measured at 480 nm (microplate reader, CLARIO star plus, BMG LABTECH) for 5 min. Autooxidation of epinephrine in the reaction mixture (without digestive fluid) was performed in each assay and obtained values were included in the calculations. The epinephrine-extinction coefficient was Ɛ = 4.02 mM^−1^ cm^−1^. The results were expressed as the production of nmol O_2_^•−^ min^−1^ mL^−1^ of digestive fluid. The measurements were conducted in three independent experiments, each in at least three biological replicates.

### 4.3. Measurement of the Free-Radical-Scavenging Capacity (DPPH) in Digestive Fluid

Total capacity of the reduction of the free radicals in the digestive fluid was determined using 2,2-diphenyl-1-picrylhydrazyl (DPPH) [62]. The digestive fluid (150 µL) was mixed with 60 μM DPPH dissolved in 100% (*w*/*v*) methanol. The reaction mixture was incubated for 15 min in darkness at room temperature. DPPH concentration was measured at 517 nm using a microplate reader (CLARIO star plus, BMG LABTECH). Antioxidant capacity was expressed as reduction of DPPH defined as ((A_0_ − A_s_)/A_0_) × 100%, where A_0_ is absorbance of a blank and A_s_ is absorbance of the sample. Experiments were conducted in four independent replicates with three repetitions in each.

### 4.4. Measurement of Phenolic Compounds (PCs) Content in Digestive Fluid

Content of total PCs in the digestive fluid was determined using the Folin–Ciocalteu reagent. The digestive fluid (730 µL) was mixed with 70 µL of the F–C reagent (MFCD00132625) and incubated for 5 min in darkness at room temperature. After incubation, 100 µL of 7.5% (*w*/*v*) sodium-carbonate solution was added into the mixture. After incubation of the mixture for 30 min in darkness at room temperature, the content of total PCs was measured at 765 nm using spectrophotometer Hitachi U-2900 (Tokyo, Japan). Blanks were prepared as described above with the replacement of a digestive fluid with distilled water. The standard curve was created using gallic acid (Sigma-Aldrich, St. Louis, MO, USA) as a standard. Total PC content was expressed as µg per pitcher^−1^ (total digestive fluid of the pitcher). Experiments were performed in four independent replicates with three repetitions in each.

### 4.5. Measurement of NO Generation in Digestive Fluid

NO generation in the digestive fluid was measured as efflux of derivatives of 20 μM 4-amino-5-methylamino-20,70-difluorofluorescein diacetate (DAF-FM DA, Cayman Chemical, Ann Arbor, MI, USA). A stock solution of DAF-FM DA was prepared according to manufacturer’s guidelines.

Into the mixture of 1.9 mL of digestive fluid and 100 µL of 100 mM Tris-HCl buffer pH 7.4 was added a 2 nM solution of DAF-FM DA (5 µL). Fluorescence was recorded for 2000 s (excitation 495 nm, emission 515 nm) at constant temperature of 23 °C using Hitachi F-2500 (Tokyo, Japan) spectrofluorometer. As the negative controls, the digestive fluid was replaced with distilled water. The combination of 2.0 mL of 10 mM Tris-HCl buffer, pH 7.4, mixed with the solution of 2 nM DAF-FM DA (5 µL) was used for 1 arbitrary unit calculation. The value of 1U describes the minimum and maximum fluorescence Δ. The results were expressed as arbitrary U per mL^−1^ of digestive fluid. The measurements were performed in 4 repetitions, and their exact reproducibility was confirmed.

### 4.6. Measurement of ONOO^−^ Generation in Digestive Fluid

ONOO^−^ generation in digestive fluid was measured using 30-(p-aminophenyl)fluorescein (APF,), following the manufacturer’s instructions. APF sensitivity is associated with the presence of ONOO^−^ but also ^•^OH and hypochlorite [63].

Fluorescence was recorded for 2000 s (excitation 490 nm, emission 515 nm) using the Hitachi F-2500 (Tokyo, Japan) spectrofluorometer. A final intensity of fluorescence was taken into calculations. The results were calculated per 1 mL^−1^ of digestive fluid and expressed in arbitrary units (U). The 1U was estimated from the result obtained for the maximal intensity of the fluorescence for the probe without APF. Measurements were conducted in 3–4 independent experiments with three repetitions in each.

### 4.7. Measurement of NO_2_^−^ Content in Digestive Fluid

The NO_2_^−^-concentration measurement was conducted using Griess reagent (modified, Sigma G4410), following the manufacturer’s instructions. Digestive fluid (1 mL) was mixed with 50% trichloric acid (2 µL), and vigorously vortexed. Transparent fluid (75 µL) was applied into the 96-well plates containing 75 µL of Griess reagent. After 15 min of incubation in darkness at room temperature, the absorbance was read at 540 nm using microplate reader (CLARIO star plus, BMG LABTECH). Determination was performed in the three independent experiments. NO_2_^−^ concentration was calculated using a standard curve prepared from sodium nitrite (NaNO_2_) dissolved in 10 mM Tris–HCl pH 7.4. Concentration of NO_2_^−^ was expressed as nmol per mL^−1^ of digestive fluid.

### 4.8. Statistics

All data were obtained in at least 3 independent experiments. The plants were selected randomly for the experiments from 55 individuals; only “aerial” traps were used. Data were analyzed using Statistica Software. Mean values were calculated and SD was provided. After one-way ANOVA, homogenous groups were evaluated using Fisher’s LSD post hoc test.

## 5. Conclusions

Pitchers of *Nepenthes* may be described as an “external stomach”, as these plants digest prey in the lumen of the traps. In our experiment, a comparison of the changes in the ROS and RNS content in the fluid of developed and fed traps point to a contribution of these molecules to the digestion process of pitcher plants. The presented data indicate that digestion is linked to the emission of NO, as the fluid from the fed-trap NO level was higher than that from the developed pitcher fluid. One of the most important sources of NO could be the acidification of NO_2_^−^, as the concentration of these ions decreased in the fed traps in comparison to the developed traps. Although the nourishment of the plant with an animal or proteins results in the enhanced emission of NO, it is associated with decreased content of ONOO^−^, probably due to the lower production of O_2_^•−^ at the same time, which may be a consequence of maximal ROS scavenging capacity at this developmental phase of the trap. In addition, because of the relatively low PCs content in the digestive fluid from the fed trap, enzymatic antioxidants could be the most important factors in the regulation of ROS levels during digestion. Thus, there is no doubt that digestion in pitcher plants is linked to ROS and RNS; however, due to the limitation of the experimental data, further investigations of the activity of enzymatic antioxidants and the concentrations of other ROS or ROS/RNS-dependent protein modifications should be carried out, in order to build up a comprehensive view of the regulation of this process.

## Data Availability

Upon request, the data will be provided by the corresponding author.
